# Coeliac disease in the COVID-19 pandemic: does HLA have a protective effect?

**DOI:** 10.1080/07853890.2022.2039955

**Published:** 2022-02-17

**Authors:** N. Greco, A. Meacci, B. Mora, A. Vestri, A. Picarelli

**Affiliations:** a Department of Translational and Precision Medicine, Sapienza University of Rome, Rome, Italy; b Department of Experimental Medicine, Sapienza University of Rome, Rome, Italy; c Department of Public Health and Infectious Diseases, Sapienza University of Rome, Rome, Rome, Italy

**Keywords:** Human leukocyte antigen (HLA), autoimmune disease, coeliac disease, SARS-CoV-2, COVID-19

## Abstract

**Background:**

The coronavirus disease 2019 (COVID-19), an acute respiratory disease caused by a novel coronavirus (SARS-CoV-2), is emerging as a worldwide public health emergency. Several scientific contributions reported the potential relevance of human leukocyte antigen (HLA) polymorphism and susceptibility to viruses, such as SARS-CoV. In our study, we examined a population of coeliac subjects presenting the HLA haplotype DQ2 and/or DQ8. Our aim was to evaluate whether HLA DQ2 and/or DQ8 haplotype play a role in SARS-CoV-2-infection. The aim was also to evaluate the difficulty in following the gluten-free diet due to all the adversities produced by the pandemic, such as the food supply disruption, and the difficulties in managing the clinical follow-up.

**Methods:**

191 consecutive coeliac patients completed a questionnaire on their current clinical status, psychological effects, and management of the gluten-free diet experienced during the COVID-19 pandemic and questions regarding possible SARS-CoV-2 infection.

**Results:**

Out of the 191 patients who participated in the study, 42 were full-blown coeliac and 149 were in remission. From the answers provided, 84.8% of patients declared that they no longer consider themselves vulnerable to COVID-19 as they suffer from coeliac disease; 94.2% of patients did not encounter any difficulties in managing the gluten-free diet or in acquiring specific foods and 64.9% of patients in our study underwent diagnostic testing for SARS-CoV-2. Out of this number, 31.5% did so due to contacts with subjects affected by COVID-19, 26.6% for work related reasons, 11.3% due to flu-like symptoms and 30.6% for other reasons. Only 5.8% of the enrolled patients received a diagnosis of COVID-19. Out of all the patients in our population who were diagnosed with COVID-19, 94.8% developed no symptoms and none of them needed hospitalization or intensive care.

**Conclusion:**

The hypothesis that the HLADQ2 and/or DQ8 haplotype plays a protective role against SARS-CoV-2 infection, as against other viral infections, is intriguingly suggestive.

**KEY MESSAGES**
- COVID-19 as a public health emergency;
- SARS-CoV-2 and possible complications in coeliac disease;
- Role of HLA DQ2 and/or DQ8 in SARS-CoV-2 infection.

## Introduction

The coronavirus disease 2019 (COVID-19), an acute respiratory disease caused by a novel coronavirus (SARS-CoV-2), is emerging as a worldwide public health emergency [1].

Coronaviruses are large RNA viruses that infect humans but also a wide range of animals [2].

COVID-19 infection is characterized by dry cough, fever, dyspnoea, fatigue, lymphopenia, arthralgia, ageusia and anosmia **[**3,4]. It also features gastrointestinal symptoms and asymptomatic infections, especially among young children [5]. In severe cases, it can produce bilateral interstitial pneumonia, responsible for acute respiratory distress syndrome, [6] and multi-organ failure, with a high risk of death [7].

Elderly and patients featuring comorbidities, particularly those affected by chronic respiratory and cardiovascular disease, diabetes, obesity, hypertension, cancer and immunosuppressed, reported the  case-fatality rate (CFR) [8,9]. The SARS-CoV-2 infection and related complications in patients with systemic autoimmune diseases could have unpredictable evolutionistic consequences [10]. Due to the current state of emergency, these patients have changed their usual behaviour by limiting the frequency of visits to doctors’ surgeries and hospitals to avoid contagion. As a result, these patients may suffer further complications from other diseases that may not be readily diagnosed and treated [11]. Furthermore, SARS-CoV-2 infection and possible complications in patients with systemic autoimmune diseases, such as coeliac disease (CD), could have unpredictable evolutionistic consequences.

Among the autoimmune diseases, CD, caused by the ingestion of wheat, rye and oat gluten and related proteins (gliadins and glutenins), is one with the highest prevalence (1–2%) of the general population. CD can manifest itself with both gastrointestinal and extraintestinal symptoms that disappear after a careful and strict gluten-free diet. The gluten-free diet, conducted throughout life, is the only effective therapy for the disappearance of symptoms and for the prevention of complications [12].

More than 95% of coeliacs have the human leukocyte antigen (HLA) DQ2 and/or DQ8 haplotype [13].

HLA is a specific group of molecules expressed on the cell surface that plays a crucial role in adaptive immunity. Their essential function is to bind and visualize pathogen-derived antigens on the cell surface and present them to the appropriate T lymphocytes, triggering an immune response. The HLA-peptide complex is then recognized by CD8 + or CD4 + T lymphocytes, which activate the immune response.

HLA genes exhibit the highest level of diversity in our genome. Such population diversity makes it possible to maximize the likelihood that some individual within the general population activates their immune systems against an emerging infection, making survival possible. An individual’s HLA genetic profile may therefore partly influence the strength of the immune response to an invading pathogen due to the distinct peptide-binding properties of the encoded HLA molecules. The different immune response to the virus could justify the differences in the symptomatologic and clinical response [14].

Several scientific contributions have reported the potential relevance of HLA polymorphism with susceptibility to viruses, such as hepatitis C virus (HCV), HIV and previous SARS-CoVs. HLA is a critical component of the viral antigen presentation pathway and plays essential roles in conferring various susceptibility to viruses [15,16].

### Objective of the study

- Our aim was to evaluate whether HLA DQ2 and/or DQ8 haplotype play a role in SARS-CoV-2 infection.

Our secondary goals were to evaluate:

- difficulties in following a gluten-free diet due to disruptions in food supply;
- difficulties due to the clinical management of the outpatient follow-up for fear of going to the hospital.

## Materials and methods

After signing a specific consent form, 191 consecutive coeliac patients in the Gastroenterology Unit – Department of Translational and Precision Medicine – of the Policlinico Umberto I in Rome), completed a questionnaire either over the phone or during the waiting time before their medical evaluation.

The questionnaire was structured in four sections:

1. Questions about patients’ current clinical status;
2. Questions about the psychological effects experienced by patients during the COVID-19 pandemic;
3. Questions related to the management of the gluten-free diet during the COVID-19 pandemic;
4. Questions regarding SARS-CoV-2patients’ infection.

Inclusion criterion:

Age ≥ 18 years

Subjects diagnosed with CD

Informed consent signed

**Figure 1.**
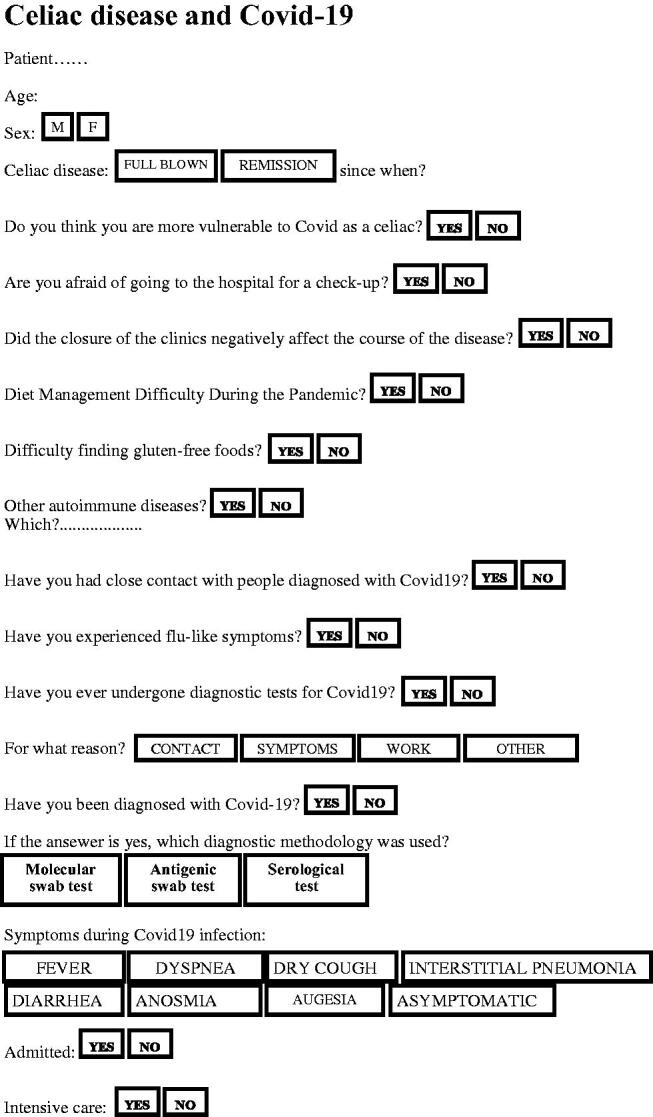
Questionnaire.

Informed consent was obtained from each patient upon entry into the study. Furthermore, all procedures were found to be in accordance with the ethical standards established by the institutional committee responsible for human experimentation (Figure 1).

## Results

Out of all patients who were asked to participate in the study, 191 met the inclusion criteria and signed informed consent.

Out of the 191 patients, 58 were males (30.4%) and 133 were females (69.6%).

The average age was 42.2 (±15.7) for male patients and 42.0 (±13.8) for female patients.

Patients with full-blown CD were 22% of the interviewees (*n* = 42), while 78% featured CD in remission (*n* = 149). There are no differences with respect to sex or with respect to undergoing investigation for COVID-19 (i.e. having active disease or in remission does not change the diagnostics for COVID-19).

From the answers provided, it emerged that 84.8% of patients declared that they no longer consider themselves vulnerable to COVID-19 as they suffer from CD and that only 28.3% were afraid of going to the hospital for a check-up visit (Table 1).

The closure of the hospitals, following the restrictions imposed due to the ongoing pandemic, did not negatively influence the course of the disease in 77.5% of patients. In addition, 94.2% of patients did not encounter any difficulties in managing the gluten-free diet or in supplying specific foods; 29.3% of the coeliac patients interviewed were affected by other autoimmune diseases and 64.9% of patients in our study population underwent diagnostic tests for SARS-CoV-2. Out of this number, 31.5% did so due to contacts with subjects affected by COVID-19, 26.6% for work related reasons, 11.3% due to flu-like symptoms and 30.6% for other reasons; 5.8% of these patients received a diagnosis of COVID-19. Out of this group, 81.8% through a molecular test and 18.2% through an antigen test. Out of all the patients in our population who received COVID-19 diagnoses, 94.8% did not develop any symptoms, while 5.2% had symptoms referable to SARS-CoV-2 infection. None of them needed hospitalization or intensive care.

## Discussion

The epidemic due to SARS-CoV-2, originated in Whuan in December 2019, spread rapidly globally assuming the features of a pandemic (World Health Organization − 11 March 2020).

The SARS-CoV-2 virus mainly and severely affects the respiratory system but also other systems can be involved such as the gastrointestinal tract, causing dry cough, fever, dyspnoea, fatigue, lymphopenia, arthralgia, diarrhoea, ageusia and anosmia.

Behavioural and management priorities, both social and economic, are changing. Some individuals have reported psychological disturbances such as states of anxiety, stress, depression, and insomnia. During the lockdown, more people increased their consumption of comfort food and reduced physical activity. Effects on the state of nutrition, which represents the first defense of the body against infectious diseases, are evident.

However, HLA also appears to play an important role in contracting the virus. HLA plays such a crucial role in the immune response to pathogens and to the development of infectious diseases, including HIV, SARS, other viruses and even malaria, hepatitis B and tuberculosis [17,18].

In our study, we examined a population of coeliac subjects presenting the HLA haplotype DQ2 and/or DQ8. Our goal was to evaluate the difficulty in adhering to the gluten-free diet due to all the adversities produced by the pandemic, such as disruptions the food supply, and difficulties in managing the clinical follow-up.

Although the COVID-19 pandemic is reported as frightening in the literature, our data show that only 15.2% our population believed to be more vulnerable and only 28.3% was afraid of going to the hospital for medical treatments and diagnostic tests. Additionally, our study shows that the examined patients had no difficulties in finding gluten-free food and therefore in following a correct gluten-free diet. This result highlights the good functioning of the specific national CD associations in offering information, fostering awareness and support to improve the quality of life of coeliac patients.

Several scientific contributions have reported the potential relevance of HLA polymorphism also in susceptibility to viruses, such as HCV or HIV and previous SARS-CoVs. The hypothesis of a protective action of the HLA haplotype DQ2 and/or DQ8 seems to be confirmed by our data, which show that only 5.8% of our small population tested positive in the tests used for the diagnosis of SARS-CoV-2. Additionally, it is worth noticing that only mild symptoms, never requiring hospitalization, have been reported by COVID-19 positive coeliac patients.

## Conclusion

In our study, we examined a population of coeliac subjects. We evaluated the difficulty of following a gluten-free diet during a pandemic. We assessed the disruptions in the food supply and the difficulties in managing the clinical follow-up. Only 15.2% of our population believed to be more vulnerable to COVID-19 and only 28.3% feared to go to the hospital for medical treatments and diagnostic tests. According to the results, the examined patients had no difficulty in finding gluten-free foods. Only 5.8% of our small population tested positive for SARS-CoV-2 diagnostic tests. Only mild symptoms, which never required hospitalization, were reported by COVID-19 positive coeliac patients.

Although a larger sample is needed, the hypothesis that the HLA DQ2 and/or DQ8 haplotype plays a protective role against SARS-CoV-2 infection, as against other viral infections, is intriguingly suggestive.

Further studies proving the existence of a functional association between HLA and COVID-19 are needed.

## Ethical approval

The study was conducted according to the guidelines of the Declaration of Helsinki, and approved by the Ethics Committee of Policlinico Umberto I Rome (study approval: Rif. 6239 – Pro0t. .9 + 0187/2021. of the Board of the Department of Translational and Precision Medicine –Sapienza University of Rome).

**Table 1.** Results of the interview carried out on all patients.

Results of the interview	
Total patients	n.191
Males	30.4 % (n.58)
Females	69.6 % (n.133)
Coeliacs in activity	22 % (n.42)
Coeliacs in remission	78% (n.149)
DQ2	14.1% (n.27)
DQ8	2.6% (n.5)
DQ2–DQ8	2.1% (n.4)
Genetics not detected	81.2% (n.155)
Patients who consider themselves more vulnerable to COVID-19	15.2% (n.29)
Patients who were afraid to go to the hospital for a check-up	28.3% (n.54)
Patients who were adversely affected by the closure of the clinics	22.5% (n.43)
Patients who have had difficulty managing the diet	5.8% (n.11)
Patients who have had difficulty searching for specific foods	9.9% (n.19)
Patients who have other autoimmune diseases	29.3% (56%)
Patients who have had contact with people who have been diagnosed with COVID-19	26.7% (n.51)
Patients who have experienced flu-like symptoms	17.3% (n.33)
Patients who have undergone diagnostic tests for COVID-19	64.9% (n.124)
They were diagnosed with COVID-19	5.8% (n.11)
COVID-19 positive patients who have had a fever	5.2% (n.10)
COVID-19 positive patients who have been hospitalized	0
COVID-19 positive patients who have been admitted to intensive care	0

## Data Availability

Data are stored in our University, Sapienza University of Rome, and are available upon request.
